# Improved biocompatibility and efficient labeling of neural stem cells with poly(L-lysine)-coated maghemite nanoparticles

**DOI:** 10.3762/bjnano.7.84

**Published:** 2016-06-27

**Authors:** Igor M Pongrac, Marina Dobrivojević, Lada Brkić Ahmed, Michal Babič, Miroslav Šlouf, Daniel Horák, Srećko Gajović

**Affiliations:** 1 Croatian Institute for Brain Research, University of Zagreb School of Medicine, Šalata 3, 10000 Zagreb, Croatia; 2 Institute of Macromolecular Chemistry, Academy of Sciences, Heyrovského Sq. 2, 16206 Prague 6, Czech Republic

**Keywords:** dextran, maghemite, nanoparticles, neural stem cells, poly(L-lysine)

## Abstract

**Background:** Cell tracking is a powerful tool to understand cellular migration, dynamics, homing and function of stem cell transplants. Nanoparticles represent possible stem cell tracers, but they differ in cellular uptake and side effects. Their properties can be modified by coating with different biocompatible polymers. To test if a coating polymer, poly(L-lysine), can improve the biocompatibility of nanoparticles applied to neural stem cells, poly(L-lysine)-coated maghemite nanoparticles were prepared and characterized. We evaluated their cellular uptake, the mechanism of internalization, cytotoxicity, viability and proliferation of neural stem cells, and compared them to the commercially available dextran-coated nanomag^®^-D-spio nanoparticles.

**Results:** Light microscopy of Prussian blue staining revealed a concentration-dependent intracellular uptake of iron oxide in neural stem cells. The methyl thiazolyl tetrazolium assay and the calcein acetoxymethyl ester/propidium iodide assay demonstrated that poly(L-lysine)-coated maghemite nanoparticles scored better than nanomag^®^-D-spio in cell labeling efficiency, viability and proliferation of neural stem cells. Cytochalasine D blocked the cellular uptake of nanoparticles indicating an actin-dependent process, such as macropinocytosis, to be the internalization mechanism for both nanoparticle types. Finally, immunocytochemistry analysis of neural stem cells after treatment with poly(L-lysine)-coated maghemite and nanomag^®^-D-spio nanoparticles showed that they preserve their identity as neural stem cells and their potential to differentiate into all three major neural cell types (neurons, astrocytes and oligodendrocytes).

**Conclusion:** Improved biocompatibility and efficient cell labeling makes poly(L-lysine)-coated maghemite nanoparticles appropriate candidates for future neural stem cell in vivo tracking studies.

## Introduction

Stem cell-based therapy is a developing area of regenerative medicine with an expected impact on the treatment of brain diseases for which there is no adequate treatment yet. Neural stem cells (NSCs) have a high self-renewal ability as well as the ability to differentiate into neurons, astrocytes and oligodendrocytes, three principal cell types of the central nervous system [1]. The transplantation of NSCs represents a possible strategy for replacing cell loss in patients suffering from different neurologic diseases such as stroke, spinal cord injury, Alzheimer’s disease or amyotrophic lateral sclerosis [2–7]. The development of non-invasive techniques to follow the stem cells through their migration, distribution, proliferation and differentiation is an essential prerequisite to characterize the biology and behavior of stem cells, to design the therapeutic approaches and minimize possible side effects [8–10]. Magnetic nanoparticles are widely used to track stem cells by magnetic resonance imaging (MRI) [11], and superparamagnetic iron oxide nanoparticles are particularly used for this purpose [12–15].

The efficient cellular uptake of nanoparticles, which would not interfere with the labeled cell activities is crucial for reliable cell tracking [16]. Biocompatible polymers are used to modify the surface of nanoparticles, prevent their agglomeration and facilitate internalization. The most widely used coating for surface modification of nanoparticles is dextran, which promotes nanoparticle internalization, in particular in different commercially available transfection agents [17]. However, the transfection methods need to be optimized for each cell line to limit cytotoxic effects of the transfection agents and increase the cellular uptake of nanoparticles [15,17]. Recent studies indicate advantages of PLL coating in comparison to dextran, since it is highly biocompatible, easy to use, available on the market and promotes internalization with highly efficiency, e.g., into human mesenchymal stem cells [18–19]. As a positively charged polypeptide, PLL is used for nonspecific adhesion of cells to solid substrates through enhancing electrostatic interaction between negatively charged ions of the cell membrane and the surface of the culture plate. Due to the presence of NH_2_ groups, which promote cell adhesion, PLL is as well used as a non-viral transfection agent for gene delivery and DNA complexation [20]. Our previous studies showed that cell labeling efficiency varied both due to the nanoparticle coating and cell type used [19,21]. Therefore NSC labeling by custom made PLL-coated nanoparticles was tested and compared to commercially available dextran-coated nanomag^®^-D-spio nanoparticles. For both types of nanoparticles the labeling efficiency, cellular viability, cytotoxicity, behavior after labeling, and the mechanism of internalization was determined and compared.

## Results

### Characterization of the nanoparticle morphology

**Figure 1:**
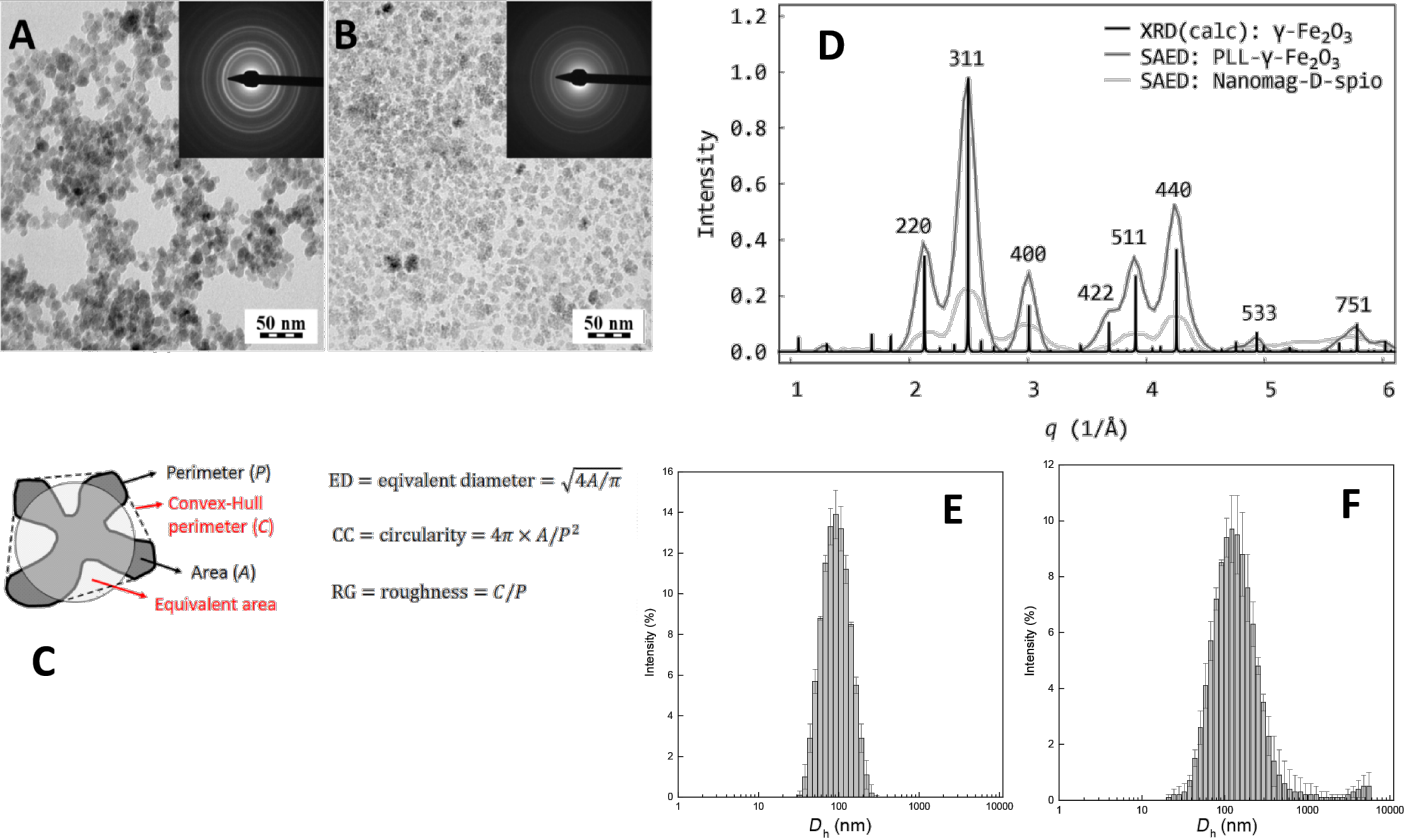
Transmission electron micrographs micrographs of (A) PLL-γ-Fe_2_O_3_ and (B) nanomag^®^-D-spio nanoparticles. Insets show the corresponding electron diffraction patterns. (C) The nanoparticle morphology was characterized by measuring morphological descriptors. Area (*A*) and perimeter (*P*) of the analyzed particles were determined by counting the pixels using an image analysis software. Convex-Hull perimeter (*C*) and equivalent area were derived auxiliary descriptors. Key morphological descriptors were equivalent diameter (ED), circularity (CC) and roughness (RG). ED determined a diameter of a circle with the same area as the measured particle. CC equaled to 1 for circles; all other shapes had CC < 1. RG of smooth objects was 1, whereas the rough objects had RG < 1. (D) Experimental selected area electron diffraction (SAED) patterns of PLL-γ-Fe_2_O_3_ and nanomag^®^-D-spio were compared to calculated X-ray diffraction (XRD) pattern of γ-Fe_2_O_3_. (E,F) Size distribution by intensity of uncoated (E) and PLL-coated (F) γ-Fe_2_O_3_ nanoparticles.

To compare the morphology of PLL-γ-Fe_2_O_3_ nanoparticles with commercially available nanomag^®^-D-spio particles, transmission electron microscopy (TEM) and dynamic light scattering (DLS) were used (Figure 1, Table 1). The average size of the PLL-γ-Fe_2_O_3_ nanoparticles (Figure 1A) was larger than that of nanomag^®^-D-spio nanoparticles (Figure 1B). The latter particles had a broader particle size distribution due to presence of tiny particles (Figure 1E,F). The smaller average particle size corresponded to low intensity diffraction rings (compare insets in Figure 1A,B). Moreover, TEM micrographs indicated different morphologies of the nanoparticles. While the PLL-γ-Fe_2_O_3_ were smooth and compact, the nanomag^®^-D-spio particles were flat with rough edges exhibiting a flake-like morphology (Figure 1). The qualitative difference between the morphologies of PLL-γ-Fe_2_O_3_ and nanomag®-D-spio nanoparticles was confirmed and quantified by image analysis showing significant differences in all measured parameters (Table 1). While TEM analysis was done on the dry particles, their hydrodynamic size in water was determined by DLS, which measured also possible particle aggregates in solution. As expected, *D*_h_ (hydrodynamic diameter obtained by DLS) was substantially larger than *D*_n_ (number-average diameter obtained by TEM) and the PLL-γ-Fe_2_O_3_ particles were larger than the nanomag^®^-D-spio particles (Table 1). However, both types of the particles were colloidally stable in water for months of storage. Presence of very small particles increased the polydispersity index PDI of nanomag^®^-D-spio (Table 1). Statistical analysis confirmed that nanomag^®^-D-spio particles were significantly less spherical and had rougher edges than PLL-γ-Fe_2_O_3_. The crystal structure of both types of the iron oxide nanoparticles was investigated using the experimental two dimensional selected area electron diffraction (SAED) patterns, which were converted to one-dimensional ones and compared with calculated X-ray diffraction patterns (XRD) of several crystalline Fe*_x_*O*_y_* forms (Figure 1). The best fit was found for γ-Fe_2_O_3_. Two facts concerning SAED patterns are worth mentioning: (i) The lower diffraction intensities of nanomag^®^-D-spio nanoparticles were in agreement with their lower average size, and (ii) since the crystal structure of Fe_3_O_4_ is close to that of γ-Fe_2_O_3_, both experimental SAED patterns are similar. As a result, the analyzed particles might be a mixture of both γ-Fe_2_O_3_ and Fe_3_O_4_.

**Table 1:** Characterization of the iron oxide nanoparticles.^a^

	*D*_n_ (nm)	*D*_w_ (nm)	PDI	*D*_h_ (nm)	*CC*	*RG*

PLL-γ-Fe_2_O_3_	10.8 ± 3	13.2	1.23	220	0.91 ± 0.05	1 ± 0.02
Nanomag^®^-D-spio	8.1 ± 4	13.0	1.61	100	0.82 ± 0.13	1.1 ± 0.04

^a^*D*_n_: number-average diameter (TEM), *D*_w_: weight-average diameter (TEM), *D*_h_: hydrodynamic diameter (DLS), PDI: polydispersity index, CC: circularity and RG: roughness.

### Cell-labeling efficiency of PLL-γ-Fe_2_O_3_ was higher than that of nanomag^®^-D-spio

**Figure 2:**
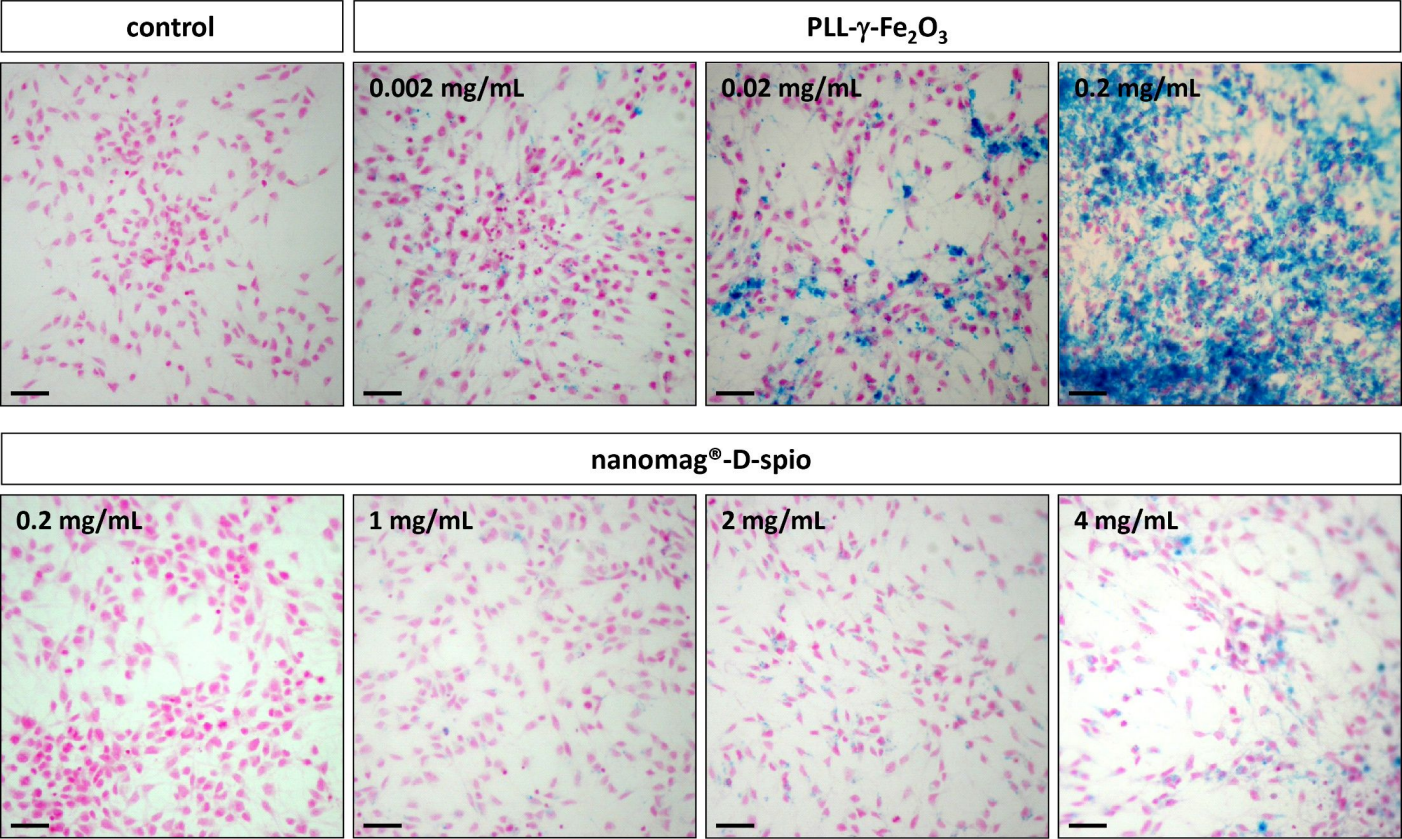
PLL-γ-Fe_2_O_3_ and nanomag^®^-D-spio nanoparticles labeling of NSCs. Light microscopy after Prussian Blue staining of NSCs labeled with different concentrations of PLL-γ-Fe_2_O_3_ (upper panel) and nanomag^®^-D-spio nanoparticles (lower panel) indicated the distribution of iron oxide nanoparticles. Nuclear Fast Red staining showed the position of nuclei. Scale bar: 50 µm.

To evaluate the uptake of nanoparticles by NSCs, Prussian blue staining was used. Both types of nanoparticles were taken up by the NSCs depending on concentration (Figure 2). When the same concentration of nanoparticles (0.2 mg/mL) was used, PLL-γ-Fe_2_O_3_-labeled cells were more intensely stained with Prussian blue than those labeled by nanomag^®^-D-spio. Considerably higher concentrations of nanomag^®^-D-spio (4.0 mg/mL) than PLL-γ-Fe_2_O_3_ (0.02 mg/mL) were needed for similar NSC cytoplasmic labeling.

**Figure 3:**
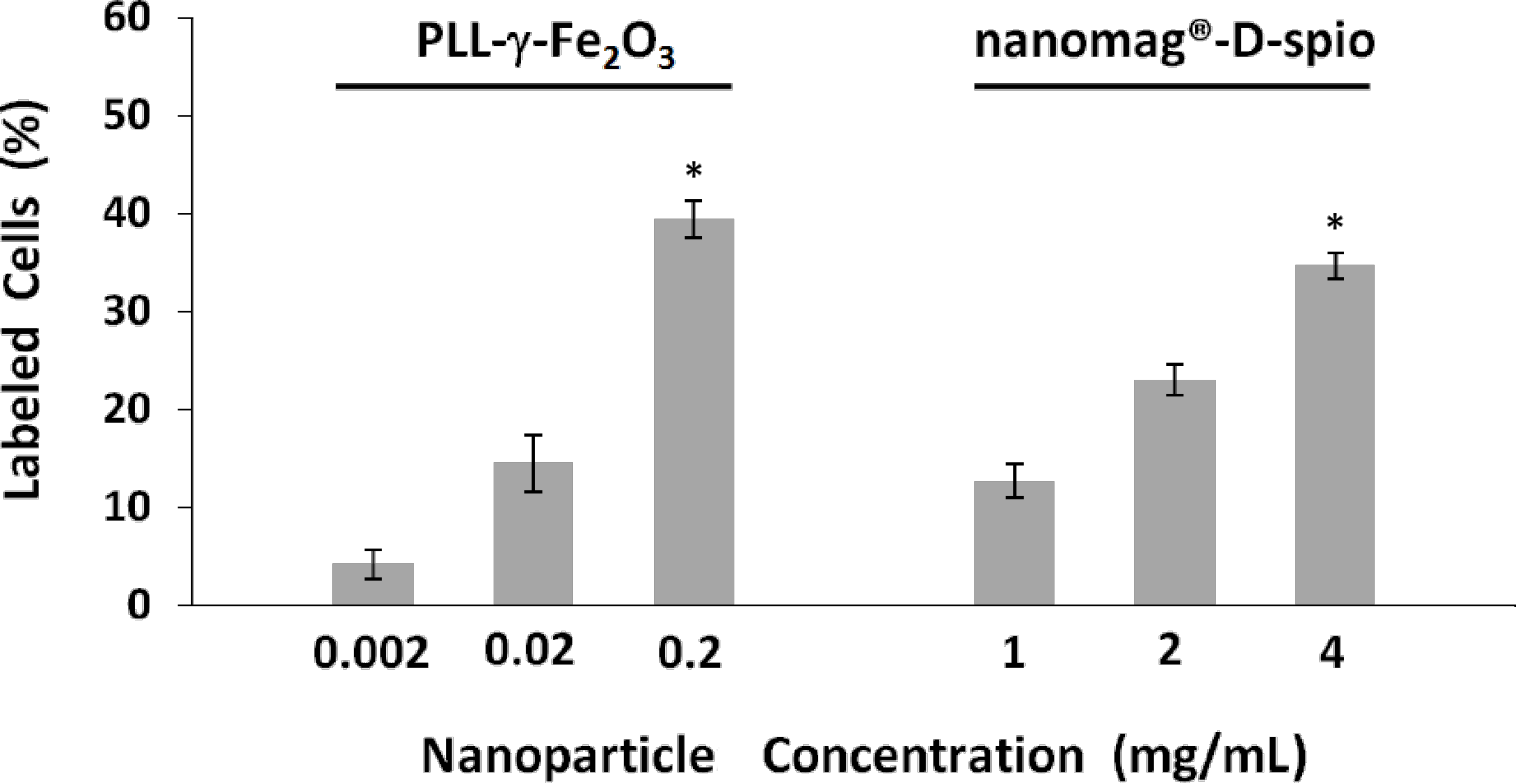
Quantitative analysis of NSC labeling of PLL-γ-Fe_2_O_3_ and nanomag^®^-D-spio nanoparticles. Overtone cumulative histogram subtraction of flow cytometry histograms of NSCs labeled with different concentrations of PLL-γ-Fe_2_O_3_ (A) and nanomag^®^-D-spio (B) nanoparticles (*N* = 5). The asterisk indicates a statistically significant difference (*P* < 0.05) versus other concentrations of the same nanoparticle.

To quantify the efficiency of cell labeling an acoustic focusing cytometer was used. The quantification of cell labeling by cytometry was considered superior to Prussian blue image quantification due to the possible adherence of stain both to the cells and to the coated dish surface. By using flow cytometry and measuring the increase of the side scattered light (SSC) of the laser beam, the intensity of which is proportional to the intracellular density and therefore reflects the nanoparticle uptake, we were able to detect the presence of nanoparticles in NSC. The percentage of nanoparticle-positive cells was determined using the Overtone cumulative histogram subtraction method (Figure 3). The labeling efficiencies for the PLL-γ-Fe_2_O_3_-labeled NSCs were as follows: (4.24 ± 1.47)% (0.002 mg/mL), (14.51 ± 2.95)% (0.02 mg/mL) and (40.34 ± 4.34)% (0.2 mg/mL). The values for the nanomag^®^-D-spio labeled NSCs were: (12.75 ± 1.72)% (1 mg/mL), (23.03 ± 1.52)% (2 mg/mL) and (38.31 ± 1.73)% (4 mg/mL; Figure 3). Similarly to Prussian blue staining, efficient labeling of PLL-γ-Fe_2_O_3_ nanoparticles was reached at the considerably lower concentration (0.2 mg/mL) compared with nanomag^®^-D-spio (4.0 mg/mL).

### Proliferation and viability

**Figure 4:**
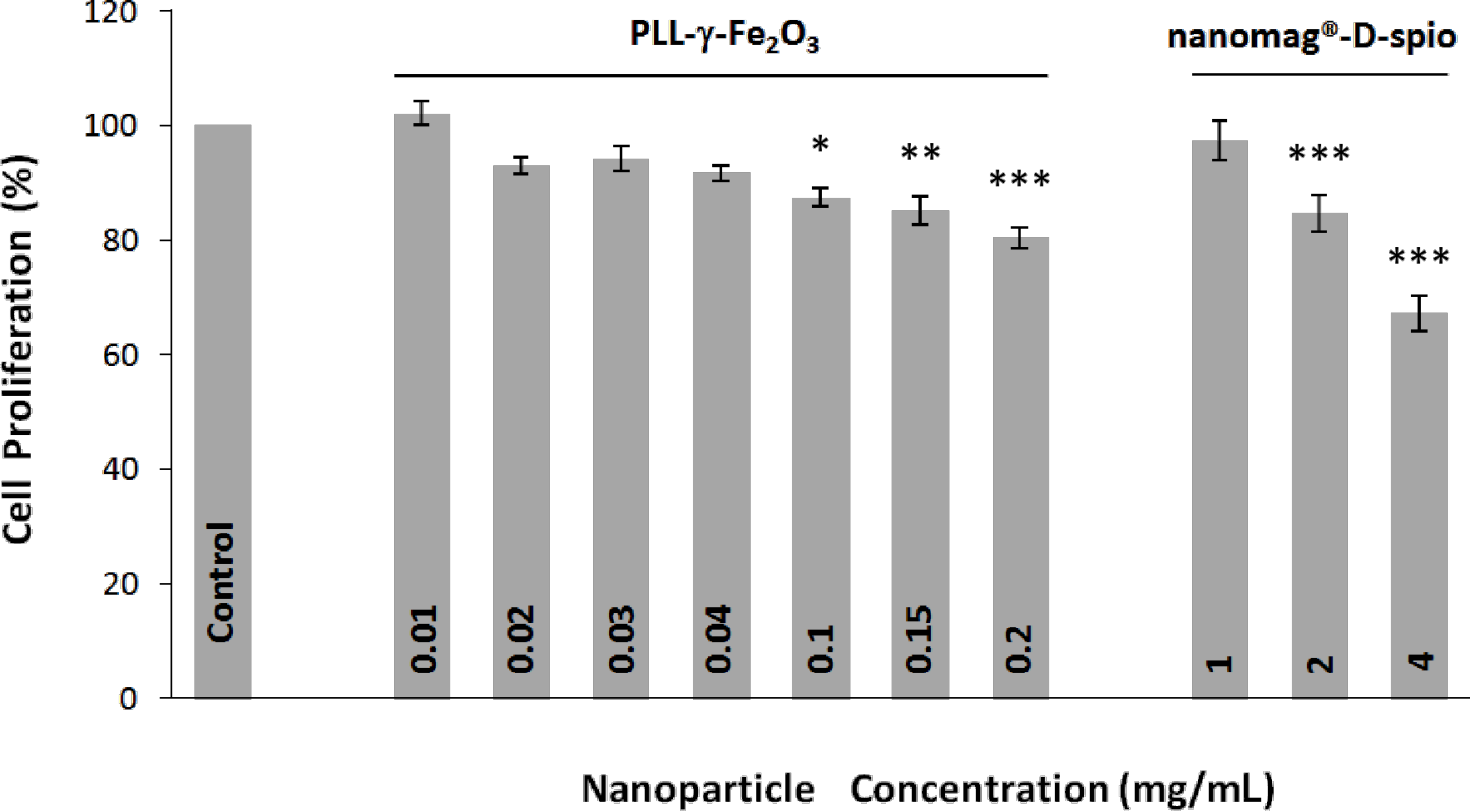
PLL-γ-Fe_2_O_3_ nanoparticles did not affect NSC proliferation. MTT cell viability assay of NSCs labeled with PLL-γ-Fe_2_O_3_ and nanomag^®^-D-spio nanoparticles (*N* = 12). The statistically significant diferences versus Control were depicted by asterisks, *: *P* < 0.05; **: *P* < 0.005; ***: *P* < 0.001.

To define if the nanoparticle labeling had any negative effect on NSC, treated cells were assessed with regard to viability, proliferation and cytotoxicity. The MTT assay was applied to demonstrate NSC viability and proliferation. A constant amount of starting cells for culture was used and compared after 48 h of NSC proliferation in the culture. The non-treated cells were considered as a standard showing viable and highly proliferated cells (100% value) and compared to the treated cells (Figure 4). For PLL-γ-Fe_2_O_3_-labeled NSCs, the values were as follows: (102.14 ± 2.04)% (0.01 mg/mL), (92.95 ± 1.41)% (0.02 mg/mL), (94.22 ± 2.18)% (0.03 mg/mL), (91.72 ± 1.37)% (0.04 mg/mL), (87.48 ± 1.69)% (0.1 mg/mL), (85.07 ± 2.43)% (0.15 mg/mL), and (80.43 ± 1.93)% (0.2 mg/mL) (Figure 4). The values for the nanomag^®^-D-spio labeled-NSCs were (97.40 ± 3.34)% (1 mg/mL), (84.63 ± 3.13)% (2 mg/mL), and (67.25 ± 3.10)% (4 mg/mL; Figure 4). When used at concentrations to achieve an efficient intracellular uptake of the nanoparticles (PLL-γ-Fe_2_O_3_ at a concentration of 0.2 mg/mL and nanomag^®^-D-spio at 4 mg/mL), PLL-γ-Fe_2_O_3_-labeled cells showed more viable cells, (80.43 ± 1.93)%, than in case of nanomag^®^-D-spio-labeled cells, (67.25 ± 3.10)%.

**Figure 5:**
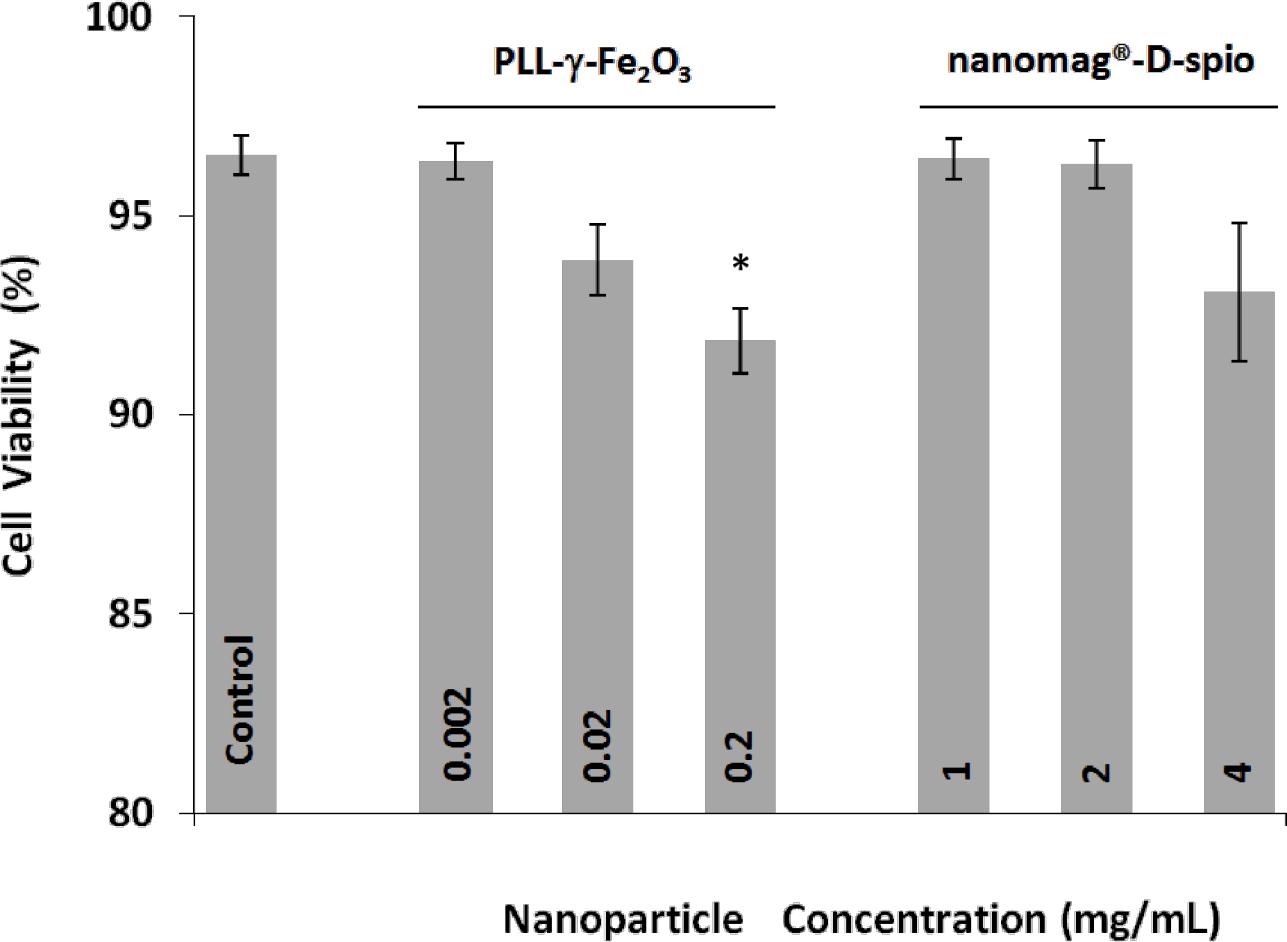
PLL-γ-Fe_2_O_3_ nanoparticles had low NSC cytotoxicity. Flow cytometry analysis showed the influence of nanoparticle cytotoxicity on the survival of NSCs labeled with PLL-γ-Fe_2_O_3_ and nanomag^®^-D-spio nanoparticles (*N* = 4). The asterisk indicates a statistically significant difference (*P* < 0.05) versus Control.

The CalceinAM/PI assay was used to assess the percentage of living cells (labeled with Calcein AM) and dead cells (labeled with PI). In contrast to the MTT assay, the obtained result was standardized on number of stained cells. The mean number of living cells in all tested conditions was higher than 90%. There was no difference between PLL-γ-Fe_2_O_3_ and nanomag^®^-D-spio, in particular when concentrations that enabled efficient labeling were considered (Figure 5).

### Both types of nanoparticles were internalized in the NSCs by actin-mediated pinocytosis

**Figure 6:**
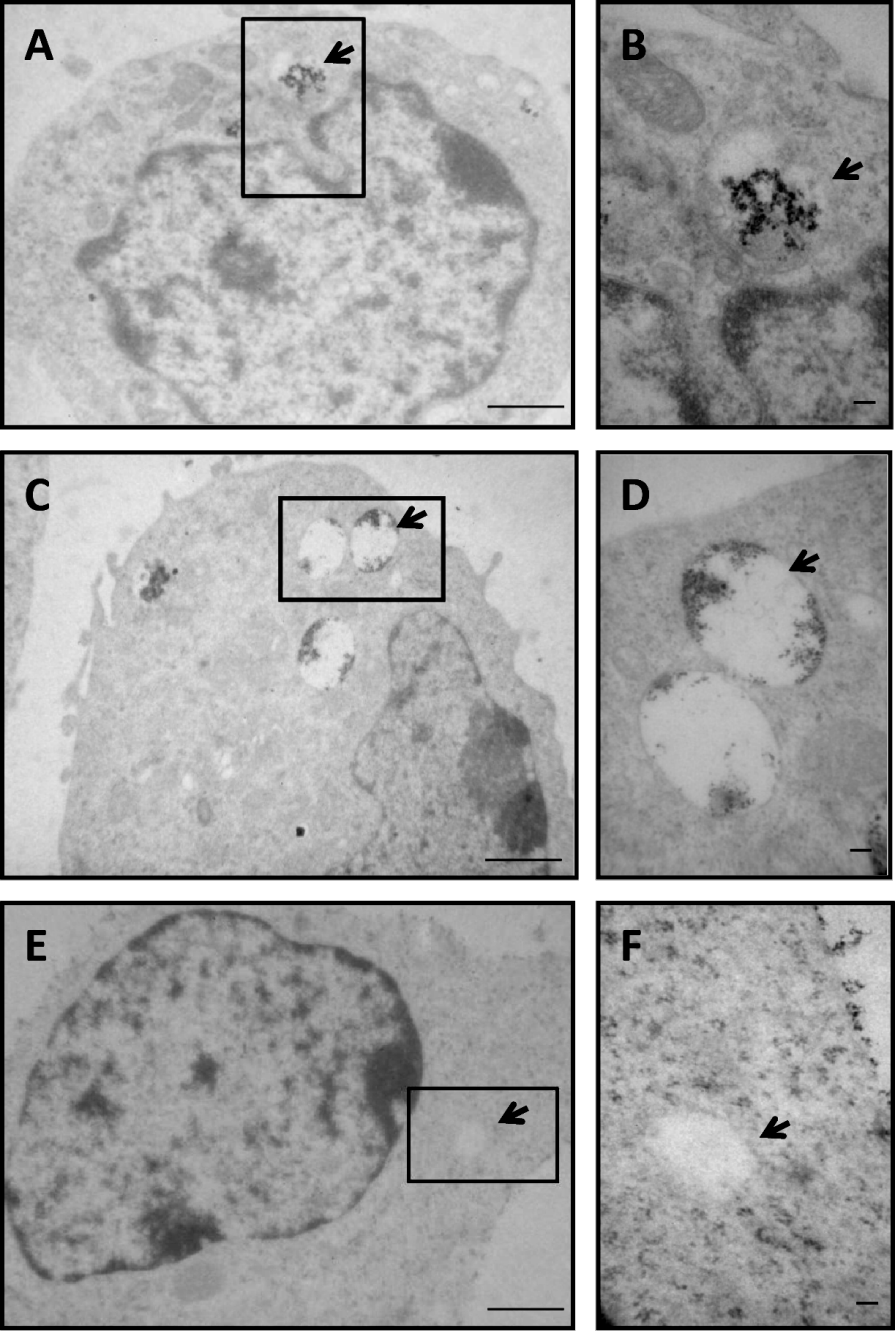
Macropinocytotic vesicle containing PLL-γ-Fe_2_O_3_ and nanomag^®^-D-spio nanoparticles. Transmission electron micrographs of NSCs labeled with PLL-γ-Fe_2_O_3_ (PLL, A, B) and nanomag^®^-D-spio (NM, C, D) nanoparticles, and unlabeled controls (E, F). Arrows indicate the macropinocytotic vesicles. Insets show macropinocytotic vesicles. Scale bar: 1 µm.

To confirm the nanoparticle internalization into NSCs, TEM was used (Figure 6). TEM micrographs demonstrated that PLL-γ-Fe_2_O_3_ or nanomag^®^-D-spio labeling did not affect the NSC ultrastructure and that both types of the nanoparticles were internalized into the cell vesicles rather than adhering to the cell surface (Figure 6).

**Figure 7:**
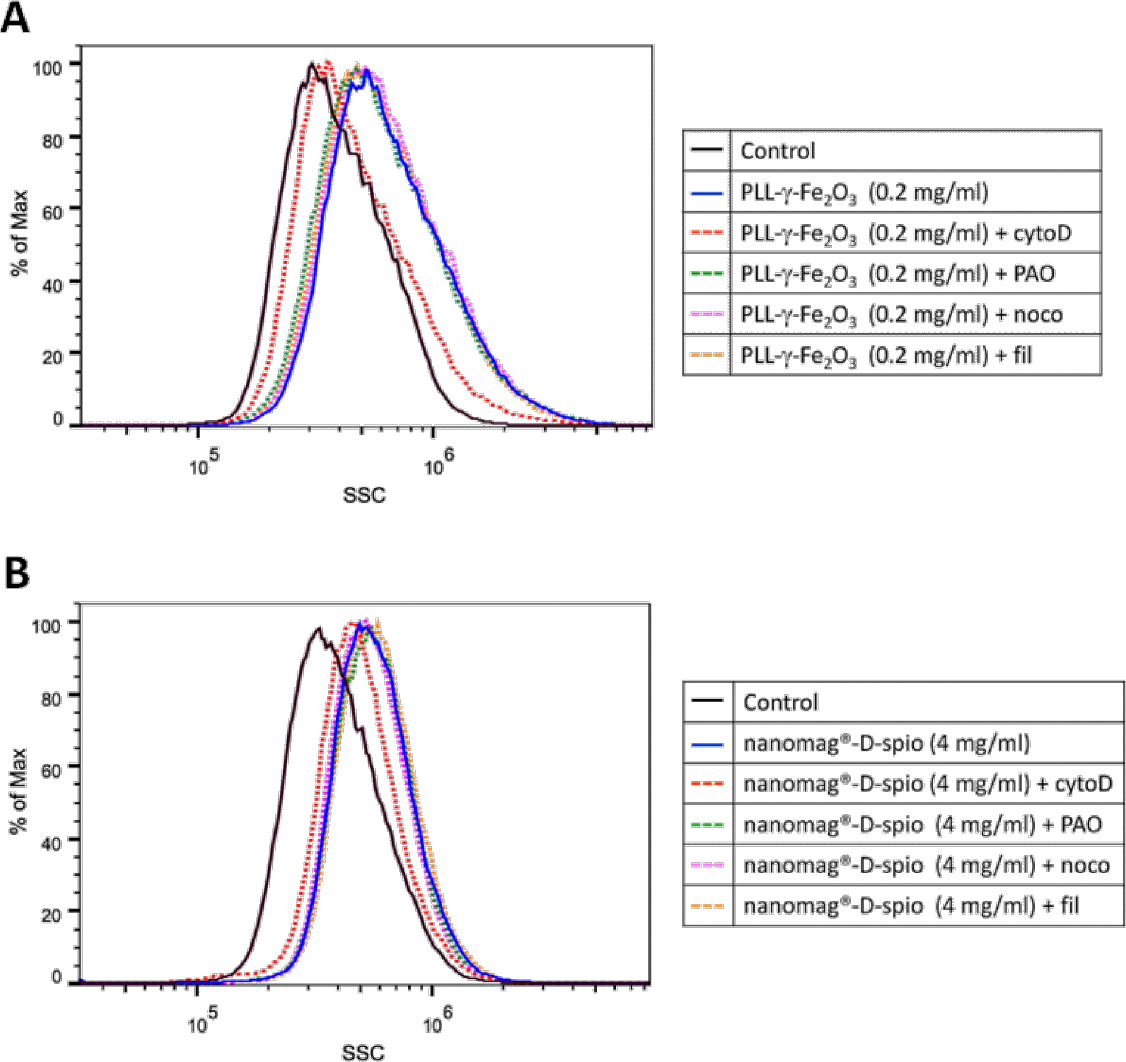
Macropinocytosis is the mechanism of cellular uptake of PLL-γ-Fe_2_O_3_ and nanomag^®^-D-spio nanoparticles. The internalization mechanism of PLL-γ-Fe_2_O_3_ (A) and nanomag^®^-D-spio (B) nanoparticles in NSCs measured by flow cytometry of side scatter (SSC) after treatment of NSCs with different inhibitors: phenylarsine oxide (PAO), cytochalasin D (cytoD), nocodazole (noco) and filipin (fil).

To determine the mechanism of the uptake of PLL-γ-Fe_2_O_3_ and nanomag^®^-D-spio nanoparticles, NSCs were treated with different endocytotic inhibitors, incubated with nanoparticles and subsequently, flow cytometry analysis of the labeled cells was performed (Figure 7A,B). The inhibitors were cytochalasine D (blocks actin-dependent process such as macropinocytosis), nocodazole (inhibits microtubule function involved in intracellular vesicle trafficking), phenylarsine oxide (inhibits the clathrin-mediated endocytotic pathway) and filipin (inhibits caveolae pathways). Flow cytometry analysis showed that NSCs treated with cytochalasine D and incubated with PLL-γ-Fe_2_O_3_ or nanomag^®^-D-spio nanoparticles exhibited a left shift in the cell granularity distribution compared with non-treated control (Figure 7). No change in labeling was observed in the phenylarsine oxide-, nocodazole- or filipin-treated NSCs when the nanoparticles were used. This indicated that actin-dependent process, e.g., macropinocytosis, was the mechanism of nanoparticle uptake for both types of nanoparticles.

### NSC progenitor post-labeling phenotype and neural differentation potential

**Figure 8:**
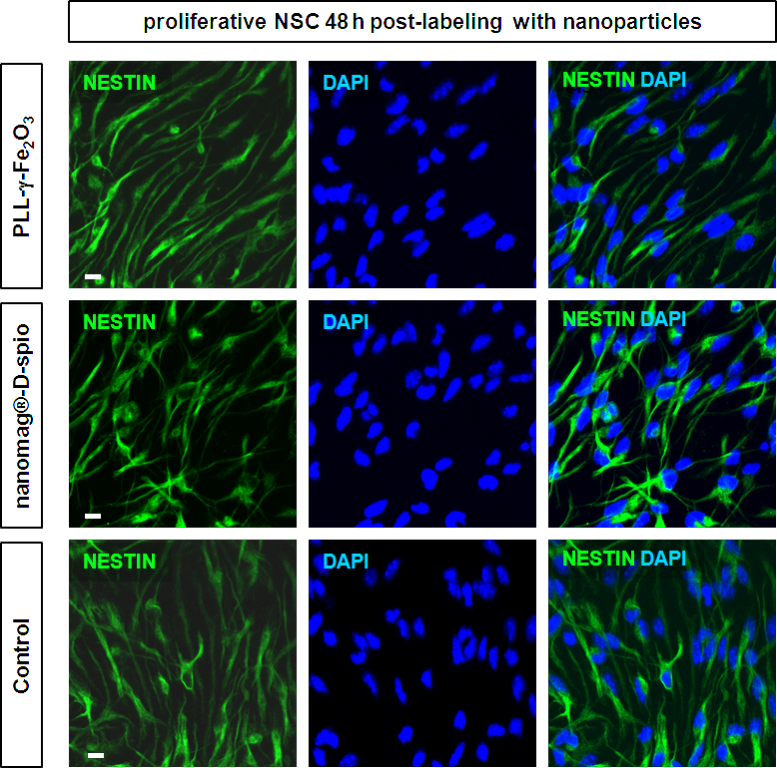
Labeling NSCs with PLL-γ-Fe_2_O_3_ and nanomag^®^-D-spio nanoparticles did not interfere with their stem/progenitor phenotype. The neural progenitor identity of NSC labeled with PLL-γ-Fe_2_O_3_ and nanomag-D-spio was confirmed by immunostaining against nestin (green), marker of neural stem cells, after 48 h of post-labeling proliferation. Control cells were not labeled with any nanoparticles. Nuclear marker DAPI was stained in blue. Scale bars: 10 µm.

**Figure 9:**
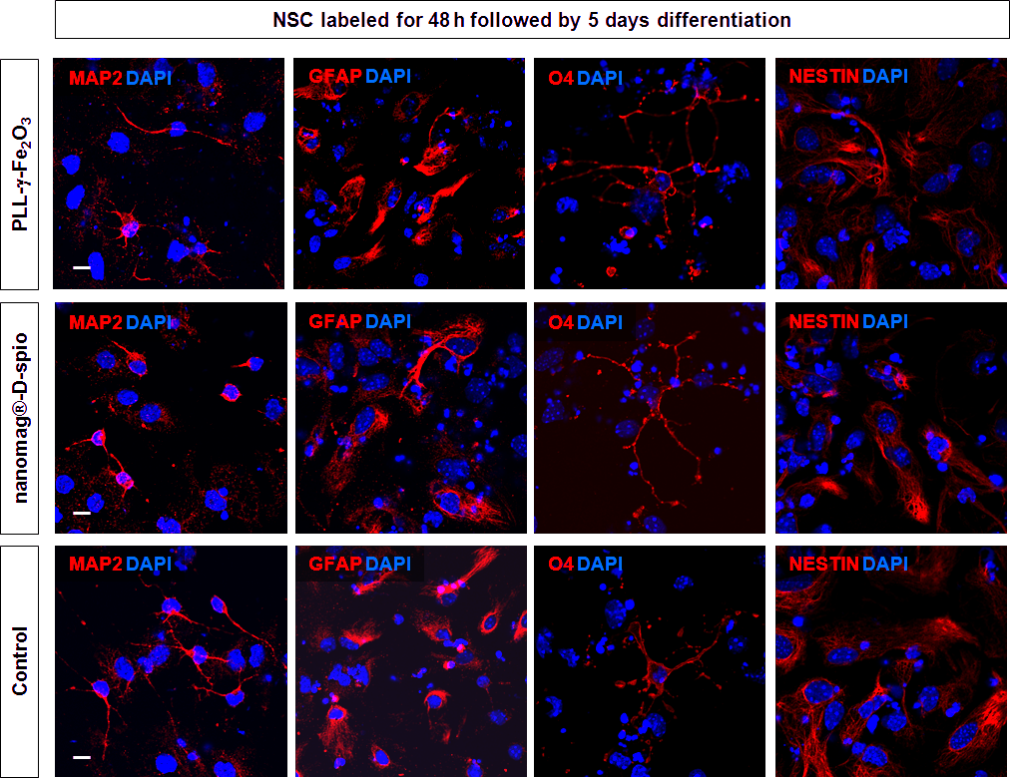
NSCs labeled with PLL-γ-Fe_2_O_3_ and nanomag^®^-D-spio nanoparticles differentiate into all three major neural cell lines. Following 48 h of nanoparticle incubation, NSCs labeled with PLL-γ-Fe_2_O_3_ or nanomag-D-spio were differentiated for 5 days and compared to the unlabeled controls. Neurons (MAP2+), astrocytes (GFAP+) and oligodendrocytes (04+) (all red) could be identified in all conditions. Nuclear marker DAPI was stained in green. Scale bars: 10 µm.

NSCs treated with PLL-γ-Fe_2_O_3_ and nanomag^®^-D-spio nanoparticles for 48 h stained positive for nestin, a marker of neural stem*/*progenitor cells, same as unlabeled control cells (Figure 8), suggesting they maintain their phenotype as neural progenitor cells. NSCs spontaneously differentiate into neurons, astrocytes and oligodendrocytes when cultured in the absence of growth factors FGF and EGF. Both PLL-γ-Fe_2_O_3_- and nanomag^®^-D-spio-labeled NSCs differentiated as the untreated controls, giving rise to neurons (MAP2+), astrocytes (GFAP+) or oligodendrocytes (O4+), when induced to differentiate for 5 days (Figure 9).

## Discussion

### PLL-γ-Fe_2_O_3_ nanoparticles as efficient tool for NSC labeling

The present study showed that PLL-γ-Fe_2_O_3_ nanoparticles were better than commercially available dextran-coated nanomag^®^-D-spio nanoparticles in NSC labeling. NSCs have a great potency to regenerate the central nervous system, and are often used as cells of choice in brain applications [2–3]. Despite a prior positive experience of using PLL-γ-Fe_2_O_3_ nanoparticles for cell labeling [22], the detailed analysis on biocompatibility of PLL-γ-Fe_2_O_3_ nanoparticles was not described, neither the method of nanoparticle cell uptake defined.

Better cellular uptake of PLL-γ-Fe_2_O_3_ nanoparticles, when compared to nanomag^®^-D-spio was similar to previous results, in which a higher particle uptake by mesenchymal stem cells in contrast to lower uptake of Endorem commercial dextran-coated nanoparticles was revealed [19]. The optimal molecular weight of PLL was necessary for obtained labeling efficiency and biocompatibility [19,21]. The optimal concentration of PLL-γ-Fe_2_O_3_ for labeling of NSCs was 0.2 mg/mL, which is considerably less than the concentration of dextran-coated nanomag^®^-D-spio (4 mg/mL) needed to achieve the same labeling efficiency. Moreover, at the optimal labeling concentration of PLL-γ-Fe_2_O_3_ nanoparticles (0.2 mg/mL), the in vitro biocompatibility was satisfactory. No detrimental effect on viability or proliferation of NSCs was observed, as compared with unlabeled control cells. Contrarily to that, the efficient concentration of nanomag^®^-D-spio (4 mg/mL) needed to achieve cell labeling, scored low (<80%) in a cell proliferation rate as compared with control unlabeled cells. To achieve the same labeling efficiency it seems that dextran-coated nanomag^®^-D-spio nanoparticles would require the addition of a transfection agent to promote internalization [23]. In addition, immunocytochemistry analysis of NSCs labeled with PLL-γ-Fe_2_O_3_ and nanomag^®^-D-spio nanoparticles suggested that they do not lose their neural stem cell identity and keep their potential to differentiate into all three major neural cell type (neurons, astrocytes and oligodendrocytes). PLL coating of nanoparticles thus provides an excellent opportunity for a safe and natural internalization of nanoparticles by NSCs.

### Macropinocytosis as a way of PLL-γ-Fe_2_O_3_ nanoparticle internalization

Proper internalization of iron particles is essential, since the particles can adhere to the cell surface thus exhibiting possible nanotoxic effects to the cell environment [24]. Endocytosis as a process of internalization of foreign materials can be divided into two major groups, phagocytosis for larger particles and pinocytosis for nanoparticles. Pinocytosis can be further subdivided depending on the size of particles into clathrin-mediated, caveolae, and macropinocytosis [25]. To determine which of the endocytotic pathway was involved in NSC uptake of PLL-γ-Fe_2_O_3_ and nanomag^®^-D-spio nanoparticles, several inhibitors related to different endocytotic pathways were tested by their incubation with NSCs prior to nanoparticle addition. These inhibitors included the inhibitor of actin-dependent process macropinocytosis cytochalasine D, inhibitor of microtubule function involved in intracellular vesicle trafficking nocodazole, inhibitor of the clathrin-mediated endocytosis phenylarsine oxide and filipin, which blocks caveolae pathways [25]. Our results suggested that the internalization of both types of the nanoparticles occurred via macropinocytosis as confirmed by TEM. Recently, PLL-γ-Fe_2_O_3_ agglomeration properties were studied in biological cell culture media with or without common serum protein, which showed the increase of size and negative ζ-potential in comparison to ultrapure water [26]. Similarly to the other studied nanoparticles, the observed changes were less pronounced in coated than in uncoated particles, and in the presence of serum protein than in its absence. Subsequently, the micropinocytosis could be the ideal cellular mechanism to internalize the range of particle sizes and properties presented to the cell in the culture conditions. Although the mechanism of nanoparticle internalization was the same for both nanoparticles analyzed, better labeling efficiency and biocompatibility makes PLL-γ-Fe_2_O_3_ nanoparticles an attractive option for future in vivo cell tracking studies.

## Conclusion

Poly(L-lysine) (PLL) polymer improved the labeling efficiency and biocompatibility of nanoparticles applied to neural stem cells (NSC). When compared to commercial dextran-coated nanomag^®^-D-spio nanoparticles, PLL-coated maghemite nanoparticles (PLL-γ-Fe_2_O_3_) excelled in labeling efficiency, viability and proliferation of NSCs without influencing their neural stem cell identity and differentiation potential. PLL-γ-Fe_2_O_3_ nanoparticles could be considered as appropriate candidates for future neural stem cell in vivo tracking studies.

## Experimental

### Nanoparticles

Nanomag^®^-D-spio was purchased from Micromod Partikeltechnologie (Rostock, Germany, catalog number 79-00-102). PLL-γ-Fe_2_O_3_ nanoparticles were prepared by chemical coprecipitation of Fe(II) and Fe(III) chlorides, oxidation with sodium hypochlorite to maghemite (γ-Fe_2_O_3_) and post-synthesis PLL coating, which was confirmed by FTIR spectroscopy [19,27]. In short, 12 mL of 0.2 M FeCl_3_ solution was mixed with 12 mL of 0.5 M NH_4_OH solution under sonication (Sonicator W-385; Heat SystemsUltrasonics, Inc., Farmingdale, NY, USA) for 2 min at room temperature to form colloid Fe(OH)_3_. Under sonication 6 mL of aqueous 0.2 M FeCl_2_ was added and the mixture poured into 36 mL of 0.5 M NH_4_OH. The formed magnetite coagulate was left to grow for 15 min, after which it was magnetically separated, repeatedly washed with ultrapure water and passed through a 0.22 µm PTFE Millex membrane filter (Millipore) to remove all impurities remaining after the synthesis. Under sonication 1.5 mL of 0.1 M sodium citrate was added, after which magnetite was oxidized by addition of 1 mL of 5% sodium hypochlorite solution. To coat nanoparticles 0.2 mL of aqueous poly(L-lysine) solution (1 mg/mL) was added dropwise with stirring to 10 mL of primary iron oxide colloid, diluted to a concentration of 2.2 mg iron oxide/mL. The obtained mixture was sonicated for 5 min and used in the experiments.

### Characterization of nanoparticles

Morphology of the particles was evaluated by transmission electron microscopy (TEM). Samples were prepared by dropping 2 μL of nanoparticle suspension on a carbon-coated copper grid. The suspension was left to equilibrate for 60 s, and water was removed by touching the bottom of the grid with a narrow strip of filtration paper. This cleaned the soluble impurities and prevented the crystallization of inorganic salts on the surface of the carbon film. The particles were dried at room temperature for more than 1 h, and TEM micrographs were obtained at an accelerating voltage of 120 kV by Tecnai Spirit G^2^ (FEI, Brno, Czech Republic). Bright field imaging (BF) and selected area electron diffraction (SAED) were used to visualize nanoparticle morphology and to identify crystal structure, respectively. Micrographs were processed by image analysis program NIS Elements (Laboratory Imaging, Prague, Czech Republic). More than 100 particles were segmented in each experiment using automated edge detection. Each particle was characterized by six morphological descriptors, namely area, perimeter, Convex-Hull perimeter, equivalent diameter, roughness and circularity (Figure 1C). To further characterize the particle size distribution, number-equivalent diameter (*D*_n_), weight-average diameter (*D*_w_) and polydispersity index (PDI) were calculated according to Equations 1–3, where *n_i_* is number of particles in class interval *i* with diameter *D_i_* [19]:

[1]
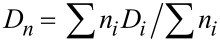


[2]
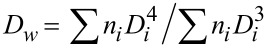


[3]
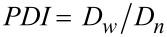


The hydrodynamic diameter (*z*-average) was determined by the cumulative analysis of time correlation functions from dynamic light scattering (DLS) using an Autosizer Lo-C (Malvern Instruments Ltd., Malvern, Great Britain). The agglomeration properties and the surface charge properties of PLL-γ-Fe_2_O_3_ nanoparticles in biological cell culture medium with and without addition of common serum protein were previously described [26].

The crystal structure of both types of nanoparticles was investigated using the experimental two dimensional selected area diffraction (SAED) patterns, which were converted to one dimension and compared to calculated X ray diffraction patterns (XRD) of several crystalline Fe*_x_*O*_y_* forms. Phase composition and size of the particles by X-ray powder diffraction was previously described [27].

### Animals

To obtain neural stem cells, wild type C57Bl/6NCrl mice were used. All animal procedures were approved by Internal Review Board of the Ethical Committee of the School of Medicine, University of Zagreb, and were in accordance with the Ethical Codex of Croatian Society for Laboratory Animal Science. All experiments were carried out in accordance with the EU Directive 2010/63/EU on the protection of animals used for scientific purposes.

### Neural stem cell culture

Neural stem cells (NSCs) were isolated from pregnant female mice as previously described [28–29]. Briefly, at gestation day 14.5, embryos were isolated and the telencephalic wall was microdissected and dissociated using StemPro Accutase (Life Technologies). Cells were maintained at 37 °C in a humidified atmosphere with 5% CO_2_/95% O_2_. Expansion medium contained DMEM/F-12 with GlutaMAX, 1% N2, 2% B27, 1% penicillin/streptomycin, epidermal growth factor (EGF) 20 ng/mL and fibroblast growth factor (FGF) 10 ng/mL (all Life technologies). The neurospheres were dissociated and plated on 24-well plates at cell density of 4 × 10^4^ NSC/well (for methyl thiazolyl tetrazolium (MTT) experiments) and 6-well plates at 2 × 10^5^ NSC/well (for Prussian blue, TEM and flow cytometry experiments). All plates were previously coated for 12 h with 50 µg/mL poly(D-lysine) (PDL) water solution (Sigma-Aldrich).

### NSC labeling with nanoparticles

Twenty-four hours after NSC plating, the nanoparticles were added directly to the culture medium and incubated for 48 h. PLL-γ-Fe_2_O_3_ nanoparticles were used in the following concentrations: 0.002, 0.01, 0.02, 0.03, 0.04, 0.1, 0.15 and 0.2 mg/mL. Nanomag^®^-D-spio nanoparticles were used in the following concentrations: 0.002, 0.02, 0.2, 1, 2 and 4 mg/mL. The nanoparticles were not added to the control (unlabeled) cells.

### Prussian blue staining

After labeling, nanoparticles were removed, cells were washed three times with phosphate buffered saline (PBS), fixed with 4% paraformaldehyde (Sigma-Aldrich) for 20 min, and stained with a 1:1 mixture of 10% K_4_Fe(CN)_6_ (Sigma-Aldrich) and 20% HCl for 20 min. Cells were counterstained with 0.1% Nuclear Fast Red (Sigma-Aldrich) for 1 min, mounted with HistoMount (Invitrogen) and covered using coverslip. After drying, the cells were analyzed under bright field using light microscope (ECLIPSE E200, Nikon Instruments, Japan).

### MTT cell viability assay

After NSC labeling MTT (methyl thiazolyl tetrazolium, 3-[4,5-dimethylthiazol-2-yl]-2,5-diphenyltetrazolium bromide) (Sigma-Aldrich) was added to the medium at a concentration of 0.5 mg/mL and incubated for 45 min at 37 °C under 5% CO_2_. The formazan crystals formed in the cells were dissolved in DMSO and the absorbance (A) was measured at 595 nm using a Microplate reader (680 XR, Bio-Rad Laboratories, Japan). MTT data were expressed as percentage of the average absorbance values of the labeled cells (sample), compared to the non-labeled cells (control) according to Equation 4:

[4]
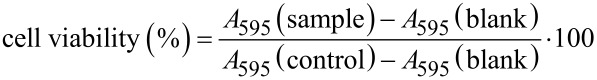


### Flow cytometry

For the nanoparticle cytotoxicity, evaluation of nanoparticle uptake efficiency and defining the mechanism of nanoparticle uptake by NSCs, an Attune^®^ acoustic focusing flow cytometer (Applied Biosystems, USA) containing a 488 nm laser, a forward-scatter (FSC) light diode detector and a photomultiplier tube of the side-scattered (SSC) light detector was used. The cytometer was set up to measure FSC linearly and SSC logarithmically. After labeling the NSCs were dissociated with StemPro Accutase (Life Technologies) cell dissociation reagent, washed with PBS, resuspended in PBS containing 2% FBS and 2 mM EDTA (pH 7.4) and passed through a 40 µm Falcon*™* cell strainer (Fisher Scientific).

To determine the nanoparticle cytotoxicity the calcein acetoxymethyl ester/propidium iodide (CalceinAM/PI) assay was applied. Dissociated cells were incubated with 0.1 µM calceinAM and 5 ng/mL PI (both Invitrogen). The percentage of alive calceinAM-positive and PI-positive NSCs was analyzed using Attune acoustic focusing cytometer and calculated using FlowJo vX.0.7 software.

To determine the nanoparticle uptake efficiency, the percentage of nanoparticle-labeled cells was determined using Attune acoustic focusing cytometer by measuring the increase of the side scattered light of the laser beam (SSC). The intensity of the SSC is proportional to the intracellular density [30]. The percentage of positive cells was determined with FCS Express 4 software (De Novo Software, Glendale, USA) using Overton cumulative histogram subtraction method [31].

To determine the mechanism of nanoparticle uptake, NSCs were pre-treated with inhibitors of endocytosis for 30 min and then incubated with nanoparticles for 48 h in the presence of the inhibitor [25]. The inhibitors were phenylarsine oxide (12 nM), cytochalasin D (60 nM), nocodazole (20 nM) and filipin (0.3 µg/mL; all from Sigma). The effect of inhibitors on cellular nanoparticle uptake was examined using an Attune acoustic focusing cytometer.

### Transmission electron microscopy of nanoparticle-labelled NSCs

After labeling, the cells were detached from the surface by 10 min treatment with StemPro Accutase reagent, washed once with DMEM/F-12 medium, separated by centrifugation and fixed overnight with 2% glutaraldehyde in 0.1 M phosphate buffer, post-fixed in 1% osmium tetroxide, and contrasted in 2% uranyl acetate in water. The samples were dehydrated in acetone and embedded in resin Durcupan (Sigma Aldrich). The ultrathin sections were cut on RMC Power Tome XL (Boeckeler Instruments, USA) ultramicrotome, contrasted with uranyl acetate and lead citrate and examined on TEM 902A (Zeiss, Oberkochen, Germany).

### Immunocytochemistry analysis

Immunocytochemistry was used to address whether labeled NSC maintain their neural progenitor phenotype as well as their neural differentiation potential. In order to confirm the progenitor identity of NSCs labeled with PLL-γ-Fe_2_O_3_ or nanomag^®^-D-spio nanoparticles, NSCs were grown on PDL/laminin-coated glass slides for 24 h, labeled with nanoparticles for 48 h and left to proliferate for additional 48 h in fresh media with FGF and EGF. In order to address their differentiation potential, NSCs were grown on PDL/laminin-coated glass slides for 24 h, labeled for 48 h with PLL-γ-Fe_2_O_3_ or nanomag^®^D-spio nanoparticles and allowed to differentiate in fresh media without of FGF and EGF for 5 days.

In both cases, NSCs were fixed in 4% paraformaldehyde for 20 min at room temperature and washed three times with PBS. Glass slides with NSCs were incubated over night at 4 °C in one of the following primary antibodies: monoclonal rat anti-Nestin (1:200; Millipore), polyclonal chicken anti-MAP2 (1:10000, Abcam), polyclonal chicken anti-GFAP (1:250; Abcam) and monoclonal mouse anti-O4 (1:50; Millipore). After washing three times with PBS, glass slides with NSCs were incubated for 2 h at room temperature with the following secondary antibodies: goat anti-mouse Alexa Flour 488 (1:500; Invitrogen), goat anti-mouse Alexa Flour 546 (1:500; Invitrogen) and goat anti-chicken Alexa Flour 546 (1:500; Invitrogen). All glass slides were counterstained with DAPI (250 ng/mL; Roche), mounted using a Fluorescence Mounting Medium (Dako) and examined using a confocal microscope (Leica SP8 X FLIM, Germany).

### Statistical analysis

For statistical analyses ANOVA with Dunnett’s method for multiple comparisons was used. Data were presented as mean values ± SEM (standard error of the mean). A probability value *P* < 0.05 was considered significant.

## References

[1] Bjornson C R, Rietze R L, Reynolds B A, Magli M C, Vescovi A L (1999). Science.

[2] Zhu J, Wu X, Zhang H L (2005). Curr Drug Targets.

[3] Lindvall O, Björklund A (2004). NeuroRx.

[4] Wang J-M, Zeng Y-S, Wu J-L, Li Y, Teng Y D (2011). Biomaterials.

[5] Cao Q, Benton R L, Whittemore S R (2002). J Neurosci Res.

[6] Park K I, Himes B T, Stieg P E, Tessler A, Fischer I, Snyder E Y (2006). Exp Neurol.

[7] Mitrečić D, Nicaise C, Gajović S, Pochet R (2010). Cell Transplant.

[8] Nagesha D, Laevsky G S, Lampton P, Banyal R, Warner C, DiMarzio C, Sridhar S (2007). Int J Nanomed.

[9] Wang L, Deng J, Wang J, Xiang B, Yang T, Gruwel M, Kashour T, Tomanek B, Summer R, Freed D (2009). Magn Reson Imaging.

[10] El-Sadik A O, El-Ansary A, Sabry S M (2010). Clin Pharmacol: Adv Appl.

[11] Himmelreich U, Hoehn M (2008). Minimally Invasive Ther Allied Technol.

[12] Sun C, Lee J S H, Zhang M (2008). Adv Drug Delivery Rev.

[13] Tseng C-L, Shih I-L, Stobinski L, Lin F-H (2010). Biomaterials.

[14] Yang H, Zhuang Y, Sun Y, Dai A, Shi X, Wu D, Li F, Hu H, Yang S (2011). Biomaterials.

[15] Li L, Jiang W, Luo K, Song H, Lan F, Wu Y, Gu Z (2013). Theranostics.

[16] Oh N, Park J-H (2014). Int J Nanomed.

[17] Arbab A S, Bashaw L A, Miller B R, Jordan E K, Bulte J W M, Frank J A (2003). Transplantation.

[18] Albukhaty S, Naderi-Manesh H, Tiraihi T (2013). Iran Biomed J.

[19] Babič M, Horák D, Trchová M, Jendelová P, Glogarová K, Lesný P, Herynek V, Hájek M, Syková E (2008). Bioconjugate Chem.

[20] Nayerossadat N, Maedeh T, Ali P A (2012). Adv Biomed Res.

[21] Babič M, Horák D, Jendelová P, Glogarová K, Herynek V, Trchová M, Likavčanová K, Lesný P, Pollert E, Hájek M (2009). Bioconjugate Chem.

[22] Babič M, Schmiedtová M, Poledne R, Herynek V, Horák D (2014). J Biomed Mater Res, Part B.

[23] Arbab A S, Jordan E K, Wilson L B, Yocum G T, Lewis B K, Frank J A (2004). Hum Gene Ther.

[24] Syková E, Jendelová P (2007). Cell Death Differ.

[25] Yang C-Y, Tai M-F, Lin C-P, Lu C-W, Wang J-L, Hsiao J-K, Liu H-M (2011). PLoS One.

[26] Domazet Jurašin D, Ćurlin M, Capjak I, Crnković T, Lovrić M, Babič M, Horák D, Vinković Vrček I, Gajović S (2016). Beilstein J Nanotechnol.

[27] Závěta K, Lančok A, Maryško M, Pollert E, Horák D (2006). Czech J Phys.

[28] Azari H, Sharififar S, Rahman M, Ansari S, Reynolds B A (2011). J Visualized Exp.

[29] Kosi N, Alić I, Kolačević M, Vrsaljko N, Jovanov Milošević N, Sobol M, Philimonenko A, Hozák P, Gajović S, Pochet R (2014). Brain Res.

[30] Zucker R M, Daniel K M (2012). Methods Mol Biol (N Y, NY, U S).

[31] Overton W R (1988). Cytometry.

